# Pooled testing for SARS‐CoV‐2 infection in an automated high‐throughput platform

**DOI:** 10.1002/jcla.23835

**Published:** 2021-06-28

**Authors:** Girish Chandra Dash, Usha Kiran Rout, Rashmi Ranjan Nanda, Debaprasad Parai, Hari Ram Choudhary, Srikanta Kanungo, Subrata Kumar Palo, Jaya Singh Kshatri, Jyotirmayee Turuk, Bijaya Kumar Mishra, Sanghamitra Pati, Debdutta Bhattacharya

**Affiliations:** ^1^ Department of Microbiology ICMR‐Regional Medical Research Centre (Dept. of Health Research, Ministry of Health & Family Welfare, Govt. of India) Bhubaneswar India

**Keywords:** Cobas 6800, high‐throughput machine, pooled testing, RT‐PCR, SARS‐CoV‐2

## Abstract

**Background:**

Active detection of SARS‐CoV‐2 infection through testing is elementary for the control of COVID‐19 pandemic. The implementation of large‐scale RT‐PCR testing has led to a rise in the demand for testing kits whose availability is always a concern.

**Objective:**

To find out the feasibility of pooled testing in a high‐throughput platform.

**Methodology:**

Pooled testing was conducted in Roche cobas 6800 in 2 methods. Firstly, the simple two‐stage testing algorithm was conducted for 1410 samples individually and then as pooled samples. Secondly, we evaluated the sensitivity of cobas 6800 for the detection of a single positive sample within a pool of negative samples.

**Results:**

Implementing the five‐sample Dorfman pooling to test 1410 samples, we identified 42 (2.9%) individual SARS‐CoV‐2‐positive samples and 27 (9.5%) positive pool samples. The pooling strategy precisely identified all the positive samples. All individually negative samples were also accurately determined by pooling. There was 100% sensitivity of detecting positive samples in a pool of negative samples even up to 1:64 dilution. There was a threefold increase in total throughput in one‐third of the cost per day.

**Conclusion:**

A high‐throughput platform such as Cobas 6800 can effectively increase the testing capacity by twofold to threefold by adopting the pooled testing strategy for successful management of SARS‐CoV‐2 and helping in the containment of community transmission.

## Graphical abstract

Five‐sample Dorfman pooling to test 1410 samples in Automated Cobas 6800 showed precise identification of all positive samples. There was 100% sensitivity of detecting positive samples in a pool of negative samples even up to 1:64 dilution. There was a threefold increase in total throughput in one‐third of the cost per day. If introduced effectively, pooling strategy would significantly help to reduce testing time, work, and reagents, enabling a substantial increase in productivity of clinical diagnostic laboratories and opening the door for the productive screening of large populations to detect the presence of SARS‐CoV‐2 infection.

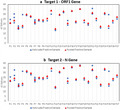

## 1 INTRODUCTION

COVID‐19 pandemic since its detection in December 2019 has raised a major concern and challenge for healthcare services and their infrastructure.^1^, ^2^ RT‐PCR–based diagnostic confirmation of infected individuals is considered to be crucial to contain viral spread because the infection can be asymptomatic despite high viral loads.^3^ It has spread rapidly to both developed and developing countries, with most countries facing acute scarcity of certain reagents that are critical for performing SARS‐CoV‐2 detection assays.^4^

There is uncertainty regarding patterns, reinfection, and vaccination of COVID‐19, which has led to an enormous demand for testing worldwide, especially in a heavily populated country such as India.^5^ Detection of new strains of SARS‐CoV‐2 has again stressed the timely and accurate testing for early detection and treatment or prevention of COVID‐19 infection in the community.^6^ India is currently 2^nd^ most affected country with more than 10 million cases with a testing capacity of around 1 million tests per day.^7^, ^8^

In view of this, we carried out a study to understand the feasibility of pooling in the high‐throughput platform and to propose a testing strategy that is simple to enforce and can extend the capacity of the existing laboratory facilities and test kits when screening a large number of individuals.

## 2 MATERIALS AND METHODS

All the samples were collected from different parts of Odisha, India, and tested at ICMR‐Regional Medical Research Centre, Bhubaneswar, using Cobas 6800 instrument. The instrument targets two genes, that is, the ORF1 gene, which is a non‐structural region that is unique to SARS‐CoV‐2 (target 1); and the E gene, which is a conserved region in the structural protein envelope for pan‐sarbecovirus detection (target 2). Cobas 6800 tests 94 samples along with 1 positive and 1 negative control with a turnaround time of 2 h 50 min per batch. A maximum of 15 batches can be run in 24 h with a maximum of 1410 samples being tested in a day. Pooling approaches were studied by 2 methods. Firstly, we adopted the simple two‐stage testing algorithm known as Dorfman pooling^9^ with minor modification to avoid selection bias. In the current study, 3 sets of 470 samples were selected and tested in 2 steps. Each set of 470 samples was tested in a single batch of 94 pools containing 5 samples each, followed by individual testing of 470 samples in 5 batches of 94 samples.

Secondly, we evaluated the sensitivity of COBAS for the detection of a single positive sample within a pool of negative samples. Five random positive samples were selected and tested in 5 different sets. In each set, a single positive sample was selected and mixed manually into pools of various sizes in ratios of 1:1, 1:2, 1:4, 1:8, 1:16, 1:32, and 1:64 using Viral Transport Medium (VTM). The single positive sample along with the 7 mixed pools was put up for testing. The total machine loading volume was 600 ul, which was equal for each sample. All statistical analyses were performed using MS‐Excel 2016. Calculations of 3‐pool and 10‐pool strategies were done according to individual positive to pool positive pattern found in the 5‐sample pool experiment. The study is approved by the institutional human ethics committee at ICMR‐Regional Medical Research Centre, Bhubaneswar.

## 3 RESULTS

**FIGURE 1:**
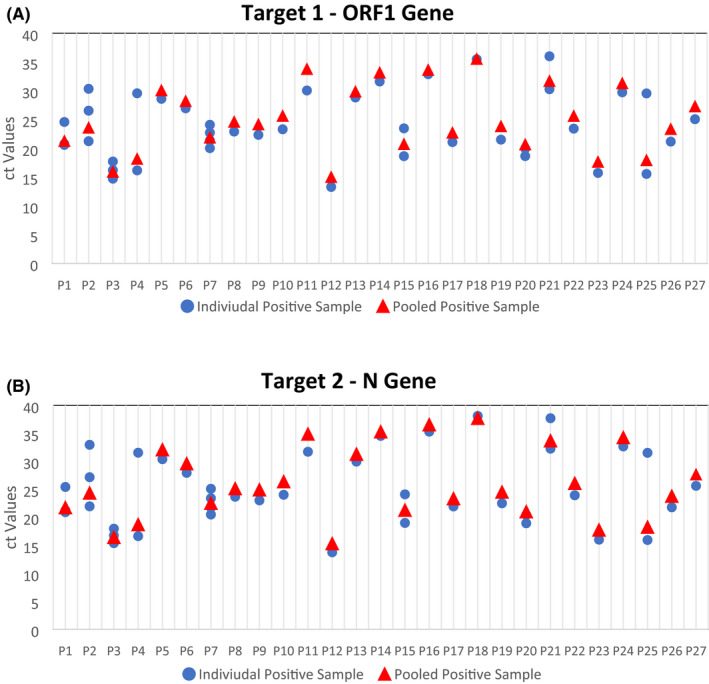
Cycle threshold (ct) values of individual positive samples vs pooled positive sample

**FIGURE 2:**
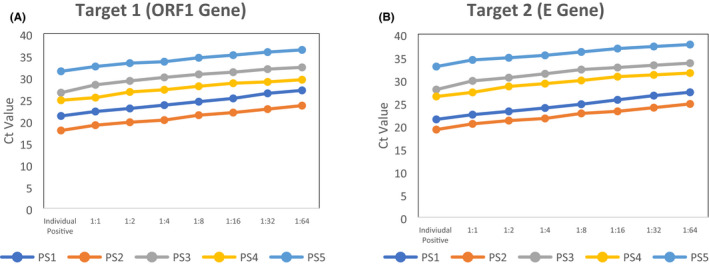
Cycle threshold (ct) values of individual positive samples in dilution up to 1:64. *PS—positive specimen

The mean age of the samples tested was 32.29 (± 15.34) years, ranging from 6 to 78 years. The male population (68.4%) was found to be significantly higher than the female population (31.6%). The samples tested were predominantly collected from asymptomatic individuals (83.6%). Out of 1410 samples tested individually, 42 samples (2.9%) were found positive. A total of 27 (9.5%) pools were found positive. The mean ct values of the individual positive samples were 23.85 (95% CI 22.06–25.65) and 24.91 (95% CI 22.90–26.92) for target 1 and target 2 genes, respectively. The mean ct value of the pooled sample was 25.08 (95% CI 22.77–27.39) and 26.10 (95% CI 23.52–28.68) for target 1 and target 2 genes, respectively (Supplementary Table 1). The ct values of the pools were found to be more than the individual positive samples where one positive sample was found except in one pool (P18). The ct value of the pool lied in between the ct values of the individual positives where multiple positives were found (Figure 1A,B). The sensitivity was 100% for detecting positive samples in the pool of negative up to 1:64 dilution. However, as the number of negative samples increases in the pool, the amplified RNA reaches the threshold later. The distribution of viral load values of the five sets of 8 samples each containing 1 individual positive sample and 7 pooled samples with different values of negative samples ranged from 17.81 to 36.27 for Target 1 (ORF 1 Gene) (Figure 2A) and from 19.14 to 37.72 for Target 2 (E Gene). (Figure 2B) (Supplementary Table 2). The cycle threshold (Ct) values were found to be in increasing order as the dilution level increases for both the targeted genes.

At the current positivity rate of 2.9%, there were a threefold rise in the number of samples tested in 5‐pool strategy and a fivefold rise in 10‐pool strategy per day with a maximum number of samples tested to be 3370, 4041, and 7618 in 3‐pool, 5‐pool, and 10‐pool strategy (Supplementary Table 3). Consequently, the cost per sample tested decreased by 1/3^rd^ and 1/5^th^ in 5‐pool and 10‐pool strategies, respectively. As the positivity rate increases, the number of samples tested and the cost per sample increased. The estimated cost of testing per sample decreased from 19.50 US$ to 6.80 US$ and 3.61 US$ in 5‐pool and 10‐pool strategy, respectively. At 25% positivity, the cost per sample tested was found to be more than the original cost. The turnaround of reporting positive samples increased to 6 h 10 min from the original 2 h 50 min after the sample has been received at the laboratory.

## 4 DISCUSSION

Comprehensive population testing policies are specifically intended to detect asymptomatic or paucisymptomatic individuals with SARS‐CoV‐2 infection, who are considered to be a major source of transmission.^10^ Shortage of trained manpower, reagent kits, and other supplementary materials could also enforce implementation of pooling strategy to increase the daily capacity of the laboratories. Routine monitoring of various crucial and high‐risk organizations such as hospital settings and military staff can also be an indication of the need for pooling. Pooling techniques for RT‐PCR testing can be beneficial if the proportion of positive specimens in the sample collection is low (~1%).^4^ The incidence of SARS‐CoV‐2 infection in the population being tested is not always understood, which may impact the determination of optimum pool size. External statistics, such as a prior analysis of individual samples, the prevalence of symptomatic patients, or alternate approaches, such as serological screening, may resolve the issue.^11^ In our study, we undertook a maximum calculation of 10‐pool strategy as the positivity rate was high. Our study showed there is an increase in productivity until 20% positivity rate after which there is a decline in the maximum number of samples tested per day. Various studies have suggested 30‐pool strategy in low prevalence areas.^12^ Our study showed that Cobas 6800 can effectively detect the positive sample in a dilution of 1:64 samples, which was the first of its kind to find out the sensitivity of Cobas 6800.

As observed in other studies with RT‐PCR machine, the cycle threshold (Ct) values were found to be in increasing order as the dilution level increases for both the targeted genes.^9^, ^10^

A study has demonstrated that optimum pool size and prevalence rates are inversely proportional; if the prevalence rate of COVID‐19 in the community is low, more samples could be pooled, which can result in different pool sizes being tested according to the level of virus circulation.^4^ Our findings were in line with other studies that suggest pooling can effectively increase the laboratory capable of testing for identifying positive samples with adequate diagnostic accuracy.^13^, ^14^

The study demonstrates the usefulness and effectiveness of pooled sampling in a high‐throughput machine such as Cobas 6800. The method's simplicity, similarities to currently approved practices, and the non‐requirement of any specific sample handling or additional in‐information make it easy to implement on a broader scale. However, manual pooling procedure has to be performed carefully with proper coding and decoding of samples, for avoiding reporting errors that could be improved by tailored tools for pooling calculation. Secondly, a higher positivity rate could affect the effective pool size required for testing, which sometimes increases the turnaround time of positive samples reporting that could affect individuals requiring emergency services. If introduced effectively, pooling strategy would significantly help to reduce testing time, work, and reagents, enabling a substantial increase in productivity of clinical diagnostic laboratories and opening the door for the productive screening of large populations to detect the presence of SARS‐CoV‐2 infection.

## CONFLICTS OF INTEREST

There are no conflicts of interest.

## AUTHORS CONTRIBUTIONS

DB & SP designed the study. DB, GCD, UKR, RRN, DP, and SK were involved in the testing and analysis of data. SKP, JSK, JT, and BM were responsible for data analysis and valuable inputs. HRC, DB, and BK did the statistical analysis. SP, DB, GCD, and UKR wrote the article. All authors read and approved the final article.

## Supporting information

Table S1‐3

## Data Availability

Data and other supplementary data are available on request.
