# Supplementary material for: Pooled testing for SARS‐CoV‐2 infection in an automated high‐throughput platform
Source: J Clin Lab Anal. 2021 Jun 28;35(7):e23835. doi: 10.1002/jcla.23835 (PMC8274989; doi:10.1002/jcla.23835)
Supplement: Supplementary file 1 — Table S1‐3 [file JCLA-35-e23835-s001.docx]

**Supplementary Tables**

**Supplementary Table 1: Detailed comparison of cycle threshold between the individuals and pooled positive samples**

| **Pool Batch No** | **No of Pools** | **No of Samples** | **No of Positive Pool** | **No of Individuals**  **Positives** | **Positive Pooled** | | | **Positive Individual** | | |
| --- | --- | --- | --- | --- | --- | --- | --- | --- | --- | --- |
|  |  |  |  |  | ***Positive Pool No*** | ***ORF1 Gene***  ***(Ct Value)*** | ***E Gene***  ***(Ct Value)*** | ***Individual Positivel No*** | ***ORF1 Gene***  ***(Ct Value)*** | ***E Gene***  ***(Ct Value)*** |
| 1 | 94 | 470 | 10 | 20 | 1 | 21.27 | 21.75 | 1 | 24.59 | 25.44 |
|  |  |  |  |  |  |  |  | 2 | 20.77 | 21.26 |
|  |  |  |  |  |  |  |  | 3 | 20.6 | 20.93 |
|  |  |  |  |  | 2 | 23.59 | 24.36 | 4 | 30.35 | 32.97 |
|  |  |  |  |  |  |  |  | 5 | 26.55 | 27.18 |
|  |  |  |  |  |  |  |  | 6 | 21.25 | 21.98 |
|  |  |  |  |  | 3 | 15.91 | 16.43 | 7 | 16.22 | 16.78 |
|  |  |  |  |  |  |  |  | 8 | 17.72 | 17.98 |
|  |  |  |  |  |  |  |  | 9 | 14.74 | 15.4 |
|  |  |  |  |  | 4 | 18.17 | 18.72 | 10 | 29.57 | 31.56 |
|  |  |  |  |  |  |  |  | 11 | 16.18 | 16.65 |
|  |  |  |  |  | 5 | 30.07 | 32.1 | 12 | 28.61 | 30.4 |
|  |  |  |  |  | 6 | 28.21 | 29.61 | 13 | 26.96 | 27.96 |
|  |  |  |  |  | 7 | 21.86 | 22.54 | 14 | 24.1 | 25.11 |
|  |  |  |  |  |  |  |  | 15 | 21.38 | 21.74 |
|  |  |  |  |  |  |  |  | 16 | 22.73 | 23.42 |
|  |  |  |  |  |  |  |  | 17 | 20.04 | 20.51 |
|  |  |  |  |  | 8 | 24.61 | 25.15 | 18 | 22.92 | 23.69 |
|  |  |  |  |  | 9 | 24.15 | 24.94 | 19 | 22.35 | 23.04 |
|  |  |  |  |  | 10 | 25.64 | 26.39 | 20 | 23.32 | 24.04 |
| 2 | 94 | 470 | 8 | 10 | 11 | 33.78 | 34.9 | 1 | 30.06 | 31.73 |
|  |  |  |  |  | 12 | 15.01 | 15.32 | 2 | 13.3 | 13.76 |
|  |  |  |  |  | 13 | 29.86 | 31.3 | 3 | 29.03 | 30.4 |
|  |  |  |  |  |  |  |  | 4 | 28.84 | 29.97 |
|  |  |  |  |  | 14 | 33.15 | 35.31 | 5 | 31.62 | 34.56 |
|  |  |  |  |  | 15 | 20.73 | 21.29 | 6 | 23.48 | 24.12 |
|  |  |  |  |  |  |  |  | 7 | 18.65 | 19.01 |
|  |  |  |  |  | 16 | 33.62 | 36.57 | 8 | 32.89 | 35.32 |
|  |  |  |  |  | 17 | 22.71 | 23.36 | 9 | 21.09 | 21.96 |
|  |  |  |  |  | 18 | 35.53 | 37.71 | 10 | 35.48 | 38.13 |
| 3 | 94 | 470 | 9 | 12 | 19 | 23.83 | 24.53 | 1 | 21.52 | 22.52 |
|  |  |  |  |  | 20 | 20.68 | 21.02 | 2 | 18.66 | 18.95 |
|  |  |  |  |  |  |  |  | 3 | 19.99 | 20.45 |
|  |  |  |  |  | 21 | 31.7 | 33.7 | 4 | 36 | 37.72 |
|  |  |  |  |  |  |  |  | 5 | 30.28 | 32.28 |
|  |  |  |  |  | 22 | 25.63 | 26.09 | 6 | 23.43 | 23.95 |
|  |  |  |  |  | 23 | 17.65 | 17.75 | 7 | 15.74 | 16.02 |
|  |  |  |  |  | 24 | 31.3 | 34.27 | 8 | 29.74 | 32.68 |
|  |  |  |  |  | 25 | 17.93 | 18.27 | 9 | 29.55 | 31.54 |
|  |  |  |  |  |  |  |  | 10 | 15.56 | 15.96 |
|  |  |  |  |  | 26 | 23.35 | 23.79 | 11 | 21.18 | 21.8 |
|  |  |  |  |  | 27 | 27.29 | 27.66 | 12 | 25.07 | 25.64 |
| Total | 282 | 1410 | 27 | 42 |  |  |  |  |  |  |

**Supplementary Table 2: Cycle threshold (ct) values of individual positive samples in dilution upto 1:64.**

| **Positive Specimen No** | **Target Gene** | **Individual**  **Positive Sample**  **(ct value)** | **Dilution of Positive Sample: Negative Samples (ct value)** | | | | | | |
| --- | --- | --- | --- | --- | --- | --- | --- | --- | --- |
|  |  |  | **1:1** | **1:2** | **1:4** | **1:8** | **1:16** | **1:32** | **1:64** |
| PS1 | ORF 1 | 21.14 | 22.16 | 22.86 | 23.62 | 24.41 | 25.16 | 26.32 | 27 |
|  | E Gene | 21.37 | 22.4 | 23.12 | 23.86 | 24.68 | 25.64 | 26.56 | 27.29 |
| PS2 | ORF 1 | 17.81 | 19.03 | 19.7 | 20.19 | 21.32 | 21.91 | 22.71 | 23.51 |
|  | E Gene | 19.14 | 20.39 | 21.1 | 21.57 | 22.67 | 23.13 | 23.93 | 24.74 |
| PS3 | ORF 1 | 26.47 | 28.27 | 29.17 | 29.97 | 30.67 | 31.17 | 31.87 | 32.27 |
|  | E Gene | 27.88 | 29.78 | 30.48 | 31.33 | 32.25 | 32.7 | 33.19 | 33.65 |
| PS4 | ORF 1 | 24.74 | 25.34 | 26.64 | 27.14 | 27.94 | 28.64 | 28.94 | 29.44 |
|  | E Gene | 26.37 | 27.27 | 28.57 | 29.18 | 29.87 | 30.7 | 31.09 | 31.49 |
| PS5 | ORF 1 | 31.37 | 32.47 | 33.24 | 33.57 | 34.47 | 35.07 | 35.77 | 36.27 |
|  | E Gene | 32.91 | 34.36 | 34.83 | 35.36 | 36.1 | 36.84 | 37.29 | 37.72 |

*PS-Positive Specimen

**Supplementary Table 3: Maximum Samples tested in 3 pooled, 5 pooled and 10 pooled pooling strategy in various positivity rate**

| Positivity Rate | 3 Pooled | | 5 Pooled | | 10 Pooled | |
| --- | --- | --- | --- | --- | --- | --- |
|  | **Maximum Samples Tested per Day** | **Cost Per Sample**  **(US$)** | **Maximum Samples Tested per Day** | **Cost Per Sample**  **(US$)** | **Maximum Samples Tested per Day** | **Cost Per Sample**  **(US$)** |
| 2.9% | 3370 | 8.16 | 4041 | 6.80 | 7618 | 3.61 |
| 5% | 2950 | 9.32 | 3884 | 7.08 | 5665 | 4.85 |
| 10% | 2273 | 12.10 | 2698 | 10.19 | 3609 | 7.62 |
| 15% | 1848 | 14.88 | 2067 | 13.30 | 2648 | 10.39 |
| 20% | 1557 | 17.66 | 1668 | 16.49 | 2091 | 13.15 |
| 25% | 1346 | 20.44 | 1404 | 19.60 | 1728 | 15.92 |

*Calculation has been done according to the maximum number of batches that can be run in 24hr period i.e., 15 batches each having 94 samples.

Original cost per sample (non-pooled) = 19.5 US$

(At the time of calculation, 1US $ = Rs 76.92 INR)
